# Evaluation of Toxicological Effects of an Aqueous Extract of Shells from the Pecan Nut* Carya illinoinensis* (Wangenh.) K. Koch and the Possible Association with Its Inorganic Constituents and Major Phenolic Compounds

**DOI:** 10.1155/2016/4647830

**Published:** 2016-07-20

**Authors:** Luiz Carlos S. Porto, Juliana da Silva, Karen Sousa, Mariana L. Ambrozio, Aline de Almeida, Carla Eliete I. dos Santos, Johnny F. Dias, Mariangela C. Allgayer, Marcela S. dos Santos, Patrícia Pereira, Alexandre B. F. Ferraz, Jaqueline N. Picada

**Affiliations:** ^1^Laboratory of Toxicological Genetics, Lutheran University of Brazil (ULBRA), Farroupilha Avenue 8001, 92425-900 Canoas, RS, Brazil; ^2^University of the Campaign Region (URCAMP), Tancredo Neves Avenue 210, 97670000 São Borja, RS, Brazil; ^3^Physics, Statistics, and Mathematics Institute, Federal University of Rio Grande (FURG), Barão do Caí 125, 95500000 Santo Antônio da Patrulha, RS, Brazil; ^4^Ion Implantation Laboratory, Physics Institute, Federal University of Rio Grande do Sul (UFRGS), Bento Gonçalves Avenue 9500, 91501970 Porto Alegre, RS, Brazil; ^5^Laboratory of Clinical Pathology, Veterinary Hospital, Lutheran University of Brazil (ULBRA), Farroupilha Avenue 8001, 92425-900 Canoas, RS, Brazil; ^6^Laboratory of Pharmacognosis and Phytochemistry, Lutheran University of Brazil (ULBRA), Farroupilha Avenue 8001, 92425-900 Canoas, RS, Brazil; ^7^Pharmacology Department, Institute of Basic Sciences of Health, Federal University of Rio Grande do Sul (UFRGS), Sarmento Leite Street 500/305, 90050-170 Porto Alegre, RS, Brazil

## Abstract

*Background.* Industrial processing of the pecan nut* Carya illinoinensis* K. Koch generated a large amount of shells, which have been used to prepare nutritional supplements and medicinal products; however, the safe use of shells requires assessment. This study evaluated the toxic, genotoxic, and mutagenic effects of pecan shell aqueous extract (PSAE) and the possible contribution of phenolic compounds, ellagic and gallic acids, and inorganic elements present in PSAE to induce toxicity.* Results.* Levels of inorganic elements like K, P, Cl, and Rb quantified using the Particle-Induced X-Ray Emission method were higher in PSAE than in pecan shells, while Mg and Mn levels were higher in shells. Mice showed neurobehavioral toxicity when given high PSAE doses (200–2,000 mg kg^−1^). The LD_50_ was 1,166.3 mg kg^−1^. However, PSAE (50–200 mg·kg^−1^) and the phenolic compounds (10–100 mg·kg^−1^) did not induce DNA damage or mutagenicity evaluated using the comet assay and micronucleus test. Treatment with ellagic acid (10–100 mg·kg^−1^) decreased triglyceride and glucose levels, while treatments with PSAE and gallic acid had no effect.* Conclusion.* Pecan shell toxicity might be associated with high concentrations of inorganic elements such as Mn, Al, Cu, and Fe acting on the central nervous system, besides phytochemical components, suggesting that the definition of the safe dose should take into account the consumption of micronutrients.

## 1. Introduction

The pecan nut shells from* Carya illinoinensis* (Wangenh.) K. Koch are generated as a byproduct of processing pecan nuts produced on an industrial scale in the southern states of Brazil. Although the shells were initially considered a waste material, their fate began to change with studies showing their nutritional value [1–3]. Higher amounts of substances with antioxidant properties, such as phenolics, tannins, and gallic and ellagic acids, are present in shells when compared with the kernels [2]. The shells have been used empirically* in natura* and in nutraceutical products against obesity and hypercholesterolemia. Pecan shells and gallic and ellagic acids have been investigated in the prevention or treatment of human diseases such as metabolic and inflammatory conditions, neurological disorders, gastric ulcers, and cancer [4–10]. However, only a few studies examined their safety and chemical constituents [11].

The present study evaluated the acute toxicity of shells from the pecan species* Carya illinoinensis* (Wangenh.) K. Koch by determining the median lethal dose (LD_50_) and analyzing behavioral, biochemical, genotoxic, and mutagenic parameters in mice. In addition, gallic and ellagic acids were evaluated for these parameters and compared with pecan shells. Inorganic elements present in pecan shells were determined. Thus, the mechanisms of toxicity, genotoxic, and mutagenic effects of shells could be discussed based on their chemical constituents.

## 2. Materials and Methods

### 2.1. PSAE Preparation

Pecans (Barton variety) were collected in Cachoeira do Sul, RS, Brazil (coordinates 30°11′39.29′′S and 52°48′1.23′′W). A voucher specimen was deposited at the Herbarium of Lutheran University of Brazil (ULBRA) (Canoas, RS, Brazil); it was recorded under number 4289 and identified by Dr. Sérgio Bordignon at ULBRA. The shells were washed and dried in an oven at 35°C with air circulation for 7 days. The dried shells (450 g) were finely milled and extracted with water by decoction at 100°C for 15 min (1/10; plant : solvent). After cooling, the filtered extract was frozen and concentrated by lyophilization to obtain a final yield of 143.1 g (31.8%, w/w) pecan shell aqueous extract (PSAE).

### 2.2. Analysis of Inorganic Elements in PSAE and in Pecan Shells

The elemental composition of the PSAE and pecan shells was determined using the Particle-Induced X-Ray Emission (PIXE) technique. Briefly, the lyophilized extracts and the same finely milled dried shells used in the preparation of PSAE were pressed into thick pellets and placed in the target holder inside the reaction chamber, which was maintained at a pressure of approximately 10^−6^ mbar. A 3 MV Tandetron accelerator was used to irradiate the target with a 2.0 MeV proton beam and an average current of 3.5 nA. The X-rays produced in the samples were detected using a Si (Li) detector with an energy resolution of approximately 160 eV at 5.9 keV. The PIXE spectra were fitted and quantified using the GUPIXWIN software package developed at the University of Guelph (Guelph, Canada) [12–14] and the results are expressed in parts per million (ppm). The analysis procedure followed the standardized protocol [15].

### 2.3. Animals

Ninety-five CF-1 male mice (*Mus musculus*) weighing 35–45 g from our breeding colony were used. Animals were housed at 22 ± 2°C, under a 12 h/12 h light/dark cycle, with 60% relative humidity, and received water and food* ad libitum*. All experimental procedures were performed in accordance with the National Institute of Health Guide for the Care and Use of Laboratory Animals and were approved by the Ethics Committee on Animal Use (CEUA) at ULBRA (protocol number 201245-P).

### 2.4. Determination of LD_50_ and Experimental Groups

To determine LD_50_, 20 animals were divided into four groups: vehicle (0.9% NaCl + 5% dimethyl sulfoxide), 500 mg kg^−1^ PSAE, 1,000 mg kg^−1^ PSAE, and 2,000 mg kg^−1^ PSAE; treatments were administered by oral gavage. Signs of toxicity, including lethargy, piloerection, and increased respiratory rate, were observed immediately after PSAE administration in all doses and persisted for 1 h. The survival rates used to calculate the LD_50_ were 100%, 60%, and 0% at doses of 500, 1,000, and 2,000 mg kg^−1^, respectively, in the first 24 h. All surviving PSAE-treated animals were euthanized by decapitation. The LD_50_ was determined using probit regression analysis with a confidence interval of 5% in the StatPlus Professional 2009 software to assess the number of deaths with each dose. Probit analysis indicated an LD_50_ of 1,166.3 mg kg^−1^ with a standard error of 304.6.

Based on LD_50_, the selected highest PSAE dose was 200 mg kg^−1^ daily for three consecutive days (totalizing 600 mg·kg^−1^, approximately 50% LD_50_), following the recommendation described elsewhere [16], to evaluate genotoxic and mutagenic activities.

The animals were treated with vehicle (*n* = 10), PSAE (50, 100, or 200 mg kg^−1^, *n* = 10 per group), ellagic acid (Merck, São Paulo, Brazil) (EA; 10, 50, or 100 mg kg^−1^, *n* = 5 per group), or gallic acid (Merck, São Paulo, Brazil) (GA 10, 50, or 100 mg kg^−1^, *n* = 5 per group) by oral gavage for three consecutive days. A positive control group treated with cyclophosphamide (Cytoxan®, Asta Medica, São Paulo, Brazil) (i.p. single dose at 25 mg kg^−1^, *n* = 5) was included in the micronucleus test. Hydrogen peroxide (*ex vivo* treatment: slides from vehicle group treated with H_2_O_2_ 0.25 mM for 5 min) was used as positive control in the comet assay. The vehicle and PSAE groups (*n* = 10 per group) were also subjected to open field studies after the first dose. Five animals per group were used to perform the comet assay, micronucleus test, and biochemical analyses.

### 2.5. Biological Samples

Peripheral blood was collected from the tail vein (approximately 50 *μ*L in 12 *μ*L heparin sodium 5,000 I.U) 24 h after the first administration to perform the comet assay; the animals were treated for two other consecutive days. Twenty-four hours after the last administration, all mice were euthanized by decapitation and blood samples were collected to perform the comet assay and biochemical analyses. Bone marrow from both femurs was collected to perform the micronucleus test.

### 2.6. Open Field Behavior and Habituation

Open field behavior was conducted in a 50 × 60 cm box with 40 cm high walls, divided into 12 identical white squares marked by black lines. Thirty minutes after the first administration of vehicle or PSAE, the mice were placed in the rear left square and allowed to explore the environment for 5 min (training session). Latency to start the locomotion, crossing of black lines, and rearing times were counted and used as measurements of motivation, locomotion, and exploration, respectively [17].

The habituation test was conducted 24 h after the first exploration; the animals were placed again in the open field box for 5 min and the number of rearing times was counted (test session). Long-term retention of habituation to a novel environment can be considered a type of learning. A decrease in rearing between the first and second exploration sessions was considered to be a measure of habituation [17].

### 2.7. Comet Assay

The alkaline comet assay was conducted as previously described [18]. Images of 100 randomly selected cells stained with silver (50 cells each from two replicate slides) from five animals per group were analyzed. To calculate a damage index (DI), cells were visually allocated into five classes according to tail size (0 = no tails and 4 = maximum-length tails), which resulted in a single DNA damage score for each sample and for each group studied. Thus, the DI of the group could range from 0 (completely undamaged = 100 cells × 0) to 400 (maximum damage = 100 cells × 4). The percent damage frequency (DF) was calculated for each sample based on the number of cells with tail versus those without tail. All slides were coded for blinded analysis.

### 2.8. Micronucleus Test in Bone Marrow

The micronucleus test was performed according to international guidelines [16]. To avoid false-negative results and to obtain a value of bone marrow toxicity, the polychromatic erythrocyte/normochromatic erythrocyte (PCE/NCE) ratio was determined in 1,000 cells. The incidence of micronuclei was observed in 2,000 PCE for each animal using bright-field optical microscopy at 1000x magnification.

### 2.9. Biochemical Assays

The animals were left to fast for 6 h before euthanasia and blood collection for biochemical analysis. Approximately 500 *μ*L of blood was collected from each animal. The samples were centrifuged at 419 ×g for 8 min. The serum obtained was frozen and sent to the Veterinary Hospital of ULBRA for analysis of cholesterol, triglycerides, glucose, and alanine aminotransferase levels using Labtest kits from Labtest Diagnóstica SA (Belo Horizonte, MG, Brazil). The tests were performed using a semiautomatic biochemical analyzer (Thermoplate, TP Basic Analyzer, Shenzhen, China).

### 2.10. Statistical Analysis

Analysis of inorganic elements was determined using Student's* t*-test and when necessary the Welch correction. Open field data are expressed as the mean ± standard error of the mean (SEM). These data were examined using a one-way analysis of variance (ANOVA) followed by Duncan's test. The habituation test results are expressed as the mean ± SEM. Comparisons between rearing in training and test sessions within the same group in the habituation experiment were conducted using a paired* t*-test. All other data are expressed as the mean ± standard deviation (SD) and were analyzed using ANOVA followed by Dunnett's test. In all comparisons, *p* ≤ 0.05 was considered statistically significant.

## 3. Results

The inorganic components found in PSAE and pecan shells (SHELL) were as follows: sodium (Na), magnesium (Mg), aluminum (Al), silicon (Si), phosphorus (P), sulfur (S), chloride (Cl), potassium (K), calcium (Ca), chromium (Cr), manganese (Mn), iron (Fe), copper (Cu), zinc (Zn), rubidium (Rb), strontium (Sr), and barium (Ba). K, P, Cl, and Rb contents were higher in PSAE than in pecan shells, while Mg and Mn levels were higher in shells (Table 1).

**Figure 1:**
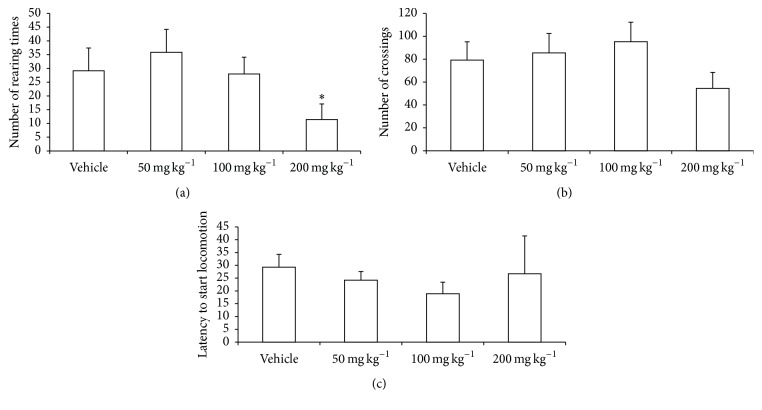
Effect of PSAE on (a) number of rearing times, (b) number of crossings, and (c) latency to start locomotion, during a 5 min exploration period in open field task (training session). Mice received vehicle or PSAE (50, 100, or 200 mg·kg^−1^) 30 min before being placed in the open field box. Data are expressed as means ± SEM. *n* = 10 animals per group. ^*∗*^ *p* < 0.05 in comparison to 50 mg·kg^−1^. ANOVA/Duncan's test.

**Figure 2:**
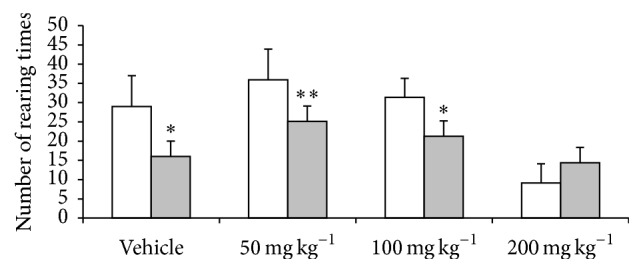
Effect of PSAE on habituation in open field (test session). The animals were placed again in the open field box for 5 min and the number of rearing times was counted. White columns: training session; gray columns: test session (measured 24 h after the training). Data are expressed as mean ± SEM. *n* = 10 animals per group; ^*∗*^ *p* < 0.05 and ^*∗∗*^ *p* < 0.01; paired *t*-test.

The behavioral pattern of the mice treated with vehicle or PSAE was evaluated using open field and habituation tasks, and the results are shown in Figures 1 and 2. As the open field test revealed, rearing (*p* = 0.469; Figure 1(a)), crossing (*p* = 0.129; Figure 1(b)), or latency to start locomotion (*p* = 0.437; Figure 1(c)) did not vary significantly between the control and test groups. However, a significant difference in rearing was observed between the mice treated with 50 and 200 mg kg^−1^ of PSAE (*p* < 0.05; Figure 1(a)).

When the animals were exposed to the open field apparatus again (24 h after training), the groups treated with 50 and 100 mg kg^−1^ as well as the vehicle group showed decreased rearing activity (Figure 2), suggesting that the animals were habituated to the environment. However, the mice treated with 200 mg kg^−1^ of PSAE showed pronounced rearing behavior when exposed to the same apparatus after 24 h, indicating that this dose may impair habituation.

The results of the comet assay performed in blood samples collected at 24 h and 72 h after treatment showed no genotoxic activities of PSAE and ellagic and gallic acids (Tables 2 and 3).

The results of the micronucleus tests (Table 4) showed no mutagenic activities of PSAE and gallic and ellagic acids at all doses tested.

There were no statistically significant differences in any biochemical parameters between the animals treated with different concentrations of gallic acid and PSAE (Table 5) and the animals in the control group (vehicle). Compared to the animals in the control group, the animals treated with different doses of ellagic acid showed a statistically significant reduction in triglyceride (49%) and glucose (47%) levels.

## 4. Discussion

Pecan nut shells from* Carya illinoinensis* (Wangenh.) K. Koch have been empirically used to decrease glucose and cholesterol levels in obese, diabetic, and/or hypercholesterolemic patients; however, few studies evaluated toxicological parameters to support their safe use as a nutritional supplement or for medicinal purpose. This study evaluated toxicity and genotoxic activities of an aqueous extract from shells (PSAE) and the involvement of their major organic and inorganic compounds that may elicit toxicological effects. In the determination of LD_50_ (1,166.3 mg kg^−1^) the toxicity and death induced by higher doses of PSAE tested in mice could be due to high concentrations of bioactive compounds, including ellagic and gallic acids. However, no toxicity signs were detected when those phenolic compounds were administered alone at doses up to 100 mg kg^−1^.

The inorganic elements present in PSAE and pecan shells were analyzed by Particle-Induced X-Ray Emission (PIXE) (Table 1). K, P, Cl, and Rb contents were higher in PSAE than in pecan shells, indicating higher bioavailability of those elements to be extracted in water during the preparation of PSAE. The same inorganic elements were found in grape juice [12] and mate tea leaves [19, 20]. This is the first report showing that inorganic elements are present in pecan shells and PSAE sourced predominantly from Barton variety trees growing in southern Brazil.

In the open field test, a single dose of PSAE (50, 100, and 200 mg kg^−1^) did not affect the locomotion and exploratory activity, in comparison to vehicle group (Figure 1). However, the animals treated with 200 mg kg^−1^ of PSAE showed decreased rearing in comparison to the group treated with 50 mg kg^−1^ (*p* < 0.05). This same highest dose impaired the habituation memory (Figure 2). Pecan shells contain organic compounds with neuroprotective effects, such as gallic acid, which attenuated the locomotor damage and brain oxidative stress induced by lead exposure in rats [21]. Ellagic acid, which is also present in pecan shells, induced neuroprotective effects against oxidative damage in diabetic rats [22]. In our previous study, high-performance liquid chromatography analysis of PSAE constituents revealed the presence of 2,690 *μ*g g^−1^ of gallic acid and 1,800 *μ*g g^−1^ of ellagic acid in acid-hydrolyzed PSAE [10]. Treatment with gallic and ellagic acids alone did not elicit behavioral impairment (data not shown), indicating that these phenolic compounds play no role in the impaired performance of the 200 mg kg^−1^ PSAE group in the open field task.

Besides phytochemical compounds, inorganic elements with recognized CNS toxicity such as Mn, Al, Cu, and Fe were found in PSAE. Aluminum has different toxic effects on cellular processes, notably on Fe homeostasis and Al deposits in the brain, and it has been the subject of several investigations because of its association with neurodegenerative diseases [23, 24]. Nevertheless, considering that the daily Al intake averages 5–10 mg, the concentration of Al in PSAE (43.7 ± 7.8 ppm) would not be sufficient to cause damage when renal function is normal, even if other Al sources are included in food and beverages [24].

The presence of Cu and Mn in PSAE (29.9 ± 14.5 ppm and 128.3 ± 7.3 ppm, resp.) is harmful, as shown in recent data that suggest that high levels of Cu and Mn are associated with brain toxicity [25–27]. The excess deposition of Mn in brain tissues, referred to as manganism, can lead to neurological abnormalities with symptoms similar to those of Parkinson's disease [28]. The tolerable daily intake of Mn for infants (60 *μ*g kg^−1^ d^−1^) was established by the World Health Organization [29]; however, factors that influence daily Mn requirements were reviewed, and the National Academy of Sciences established an adequate intake of 2.3 and 1.8 mg d^−1^ for adult men and women, respectively, and 1.2 to 1.5 mg d^−1^ for children between 1 and 8 years of age [30].

The Cu content of 29.9 ± 14.5 ppm in PSAE is higher than that previously reported in studies of other medicinal plants, fruits, and juices [19, 31]. Cu is used as a biomonitor for air pollution in plants, such as* Baccharis* sp., when exposed to polluted areas [32]. The excess intake of Cu may lead to neuropsychiatric symptoms such as those observed in Wilson's disease [33]. Oxidative stress biomarkers and antioxidative enzyme activity increased after Cu overload in male Wistar rats, reflecting Cu-induced oxidative damage [34], and daily administration of 0.15 mg Cu/100 g BW for 90 days impaired spatial memory and neuromuscular coordination, indicative of chronic Cu toxicity [35]. Thus, the effects of excess Cu, Mn, and other mineral elements on the CNS may have contributed to the observed toxicity of PSAE doses exceeding 200 mg kg^−1^. However, other chemical components might be involved in inducing toxicity. PSAE is a complex mixture of organic and inorganic compounds that interact with one other and might produce synergistic and additive effects.

PSAE, gallic acid, and ellagic acid did not increase micronucleus frequency in the bone marrow of the mice, indicating that they did not induce clastogenicity (chromosome breakage) and aneugenicity (chromosome lagging due to dysfunction of the mitotic apparatus) [36], which can lead to chromosomal mutations. Similarly, PSAE did not increase micronucleus frequency in bone marrow of Wistar rats [11]. The comet assay showed that PSAE and gallic and ellagic acids did not cause DNA damage like single and double strand DNA breaks, increased number of alkali-labile sites, or DNA-DNA and DNA-protein cross links, suggesting that they are not likely to have genotoxic effects. Moreover, the concentration of potentially genotoxic or mutagenic minerals such as Cu and Fe in PSAE is lower than the genotoxic and mutagenic doses that were determined in mice treated daily with 33.2 or 8.5 mg kg^−1^ of Fe or Cu, respectively, for six days [37].

Treatment with PSAE or gallic acid did not affect any of the biochemical parameters tested (Table 5). However, compared to the control group animals, the mice treated with ellagic acid showed a statistically significant reduction in triglyceride and glucose levels. Ellagic acid causes reversed high-carbohydrate and high-fat diet-induced symptoms of metabolic syndrome in rats [38]. This compound also inhibited porcine pancreatic lipase activity, indicating its effects on metabolism [39]. Based on these findings, studies were conducted to evaluate the efficacy of PSAE to decrease glucose, triglycerides, and cholesterol using diabetes and hyperlipidemia models in rats and the results are promising [40].

## 5. Conclusions

The pecan shells analyzed provide a range of essential micronutrients and contain high levels of antioxidants; however, the shells have inorganic elements whose accumulation can be harmful and should be taken into account in the manufacturing of nutraceutical products of* Carya illinoinensis*.

## Figures and Tables

**Table 1:** Concentration of the inorganic elements in pecan shell aqueous extract (PSAE) and pecan shells (SHELL) by PIXE (mean ± standard deviation).

Inorganic elements	PSAE (ppm ± SD)	SHELL (ppm ± SD)
K	13,658.7 ± 217.6^*∗∗∗*^	3,889.0 ± 1862.3
Na	1,159.3 ± 75.0	791.1 ± 169.9
Ca	1,146.7 ± 55.7	6,830.0 ± 3557.1
Mg	645.2 ± 137.5	1,831.0 ± 926.1^*∗*^
P	489.2 ± 44.8^*∗*^	267.8 ± 126.5
Cl	403.4 ± 13.1^*∗∗∗*^	98.9 ± 48.4
S	291.5 ± 3.4	370.6 ± 179.1
Si	214.0 ± 13.0	179.2 ± 90.8
Fe	197.4 ± 35.9	205.8 ± 108.2
Mn	128.3 ± 7.3	319.4 ± 150.5^*∗∗∗*^
Rb	54.3 ± 7.0^*∗*^	18.3 ± 9.00
Al	43.7 ± 7.8	90.1 ± 46.3
Cu	29.9 ± 14.5	15.2 ± 7.4
Ba	20.1 ± 6.1	66.7 ± 33.7
Zn	19.2 ± 8.1	19.1 ± 9.0
Sr	12.2 ± 3.5	15.2 ± 7.3
Cr	4.5 ± 0.2	2.2 ± 1.2

^*∗*^ *p* < 0.05 and ^*∗∗∗*^ *p* < 0.001. Significant difference between PSAE and SHELL (Student's *t*-test).

**Table 2:** Evaluation of the genotoxic activity of pecan shell aqueous extract (PSAE) using comet assay in blood tissue of mice.

Sampling schedule	Treatment group	DI (mean ± SD)	DF (mean ± SD)
24 h	Vehicle^a^	23.2 ± 5.0	11.6 ± 4.8
	PSAE 50 mg kg^−1^	22.5 ± 11.8	9.2 ± 3.6
	PSAE 100 mg kg^−1^	23.6 ± 7.4	12.8 ± 3.6
	PSAE 200 mg kg^−1^	34.5 ± 9.0	17.0 ± 9.5

72 h	Vehicle	24.0 ± 5.2	11.8 ± 3.6
	PSAE 50 mg kg^−1^	27.4 ± 14.5	16.6 ± 12.6
	PSAE 100 mg kg^−1^	35.0 ± 20.3	21.5 ± 14.0
	PSAE 200 mg kg^−1^	28.2 ± 13.7	15.5 ± 9.8
	Positive control^b^	234.1 ± 62.2^*∗∗∗*^	92.6 ± 9.8^*∗∗∗*^

^a^NaCl 0.9% + DMSO 5% (dimethyl sulfoxide).

^b^Hydrogen peroxide 0.25 mM (*ex vivo* treatment: slides from vehicle group treated for 5 min with H_2_O_2_ 0.25 mM).

DI: damage index, from zero (no damage, 0 × 100 cells) to 400 (with maximum damage, 4 × 100).

DF: damage frequency, calculated based on the number of cells with damage versus those without damage.

^*∗∗∗*^ *p* < 0.001: significant difference in comparison with the vehicle group (ANOVA, Dunnett's test).

**Table 3:** Evaluation of the genotoxic activity of ellagic acid (EA) and gallic acid (GA) using comet assay in blood tissue of mice.

Sampling schedule	Treatment group	DI (mean ± SD)	DF (mean ± SD)
24 h	Vehicle^a^	53.6 ± 16.9	45.8 ± 11.1
	Ellagic acid		
	EA 10 mg kg^−1^	54.5 ± 15.9	24.2 ± 3.6
	EA 50 mg kg^−1^	50.2 ± 6.8	26.5 ± 3.6
	EA 100 mg kg^−1^	51.8 ± 4.6	21.6 ± 9.5
	Gallic acid		
	GA 10 mg kg^−1^	65.4 ± 19.4	37.8 ± 14.4
	GA 50 mg kg^−1^	64.7 ± 16.7	30.7 ± 18.8
	GA 100 mg kg^−1^	40.2 ± 13.6	22.7 ± 10.6

72 h	Vehicle	55.8 ± 16.3	24.8 ± 5.6
	Ellagic acid		
	EA 10 mg kg^−1^	58.6 ± 17.3	26.8 ± 5.2
	EA 50 mg kg^−1^	60.8 ± 16.4	26.0 ± 6.1
	EA 100 mg kg^−1^	41.0 ± 5.8	19.0 ± 2.5
	Gallic acid		
	GA 10 mg kg^−1^	58.4 ± 10.2	24.6 ± 4.5
	GA 50 mg kg^−1^	31.5 ± 5.7	17.0 ± 2.2
	GA 100 mg kg^−1^	50.6 ± 5.0	21.6 ± 3.0
	Positive control^b^	254.7 ± 55.2^*∗∗∗*^	96.0 ± 6.4^*∗∗∗*^

^a^NaCl 0.9% + DMSO 5% (dimethyl sulfoxide).

^b^Hydrogen peroxide 0.25 mM (*ex vivo* treatment: slides from vehicle group treated for 5 min with H_2_O_2_ 0.25 mM).

DI: damage index, from zero (no damage, 0 × 100 cells) to 400 (with maximum damage, 4 × 100).

DF: damage frequency, calculated based on the number of cells with damage versus those without damage.

^*∗∗∗*^ *p* < 0.001: significant difference in comparison with the vehicle group (ANOVA, Dunnett's test).

**Table 4:** Mutagenic activity of pecan shell aqueous extract (PSAE), ellagic acid (EA), and gallic acid (GA) evaluated using micronucleus test in bone marrow of mice.

Treatment group	MNPCE^a^ in 2,000 PCE	PCE/NCE ratio^b^
	Mean ± SD	Mean ± SD
Vehicle	3.8 ± 1.3	1.3 ± 0.9
Positive control^c^	10.7 ± 2.5^*∗∗*^	1.0 ± 0.3

PSAE 50 mg kg^−1^	1.0 ± 1.1	1.8 ± 0.3
PSAE 100 mg kg^−1^	3.2 ± 1.6	2.2 ± 1.6
PSAE 200 mg kg^−1^	4.7 ± 2.5	1.8 ± 0.6

EA 10 mg kg^−1^	4.0 ± 0.0	1.7 ± 0.3
EA 50 mg kg^−1^	4.6 ± 2.3	1.6 ± 0.2
EA 100 mg kg^−1^	1.6 ± 1.2	1.6 ± 0.4

GA 10 mg kg^−1^	5.5 ± 1.9	2.5 ± 0.9
GA 50 mg kg^−1^	2.7 ± 1.0	1.2 ± 0.2
GA 100 mg kg^−1^	2.6 ± 0.9	1.4 ± 0.3

^a^MNPCE: micronucleus in polychromatic erythrocytes.

^b^PCE/NCE ratio: polychromatic erythrocytes/normochromatic erythrocytes ratio.

^c^Cyclophosphamide 25 mg kg^−1^.

^*∗∗*^ *p* < 0.01: significant difference in comparison with the vehicle group (ANOVA, Dunnett's test).

**Table 5:** Biochemical analyses from mice treated with pecan shell aqueous extract (PSAE), ellagic acid (EA), and gallic acid (GA).

Treatment group	Cholesterol (mg/dL)	Triglycerides (mg/dL)	Glucose (mg/dL)	ALT (UI/L)
Vehicle	111.1 ± 24.5	94.8 ± 24.2	98.1 ± 34.3	50.6 ± 28.7

PSAE 50 mg kg^−1^	121.0 ± 4.8	73.2 ± 48.3	55.3 ± 38.6	73.1 ± 25.5
PSAE 100 mg kg^−1^	147.4 ± 18.7	102.8 ± 69.0	95.7 ± 53.7	70.1 ± 10.8
PSAE 200 mg kg^−1^	142.5 ± 13.9	59.6 ± 16.6	58.1 ± 15.6	77.6 ± 43.2

EA 10 mg kg^−1^	132.4 ± 38.7	48.5 ± 12.4^*∗*^	59.6 ± 12.8^*∗*^	33.7 ± 10.2
EA 50 mg kg^−1^	142.5 ± 67.6	53.8 ± 14.2^*∗*^	51.6 ± 13.2^*∗*^	31.8 ± 7.2
EA 100 mg kg^−1^	156.8 ± 73.2	48.1 ± 19.1^*∗*^	57.6 ± 21.8^*∗*^	41.5 ± 11.6

GA 10 mg kg^−1^	137.4 ± 17.2	105.9 ± 41.4	86.9 ± 20.7	42.6 ± 19.0
GA 50 mg kg^−1^	120.6 ± 10.6	144.4 ± 20.2	142.7 ± 39.9	43.5 ± 1.8
GA 100 mg kg^−1^	133.8 ± 25.2	112.0 ± 30.2	96.1 ± 27.0	74.8 ± 61.0

Values are presented as mean and standard deviation.

^*∗*^ *p* < 0.05: significant difference in comparison with the vehicle group (ANOVA, Dunnett's test).
